# Fast Monitoring of Indoor Bioaerosol Concentrations with ATP Bioluminescence Assay Using an Electrostatic Rod-Type Sampler

**DOI:** 10.1371/journal.pone.0125251

**Published:** 2015-05-07

**Authors:** Ji-Woon Park, Chul Woo Park, Sung Hwa Lee, Jungho Hwang

**Affiliations:** 1 School of Mechanical Engineering, Yonsei University, Seoul, Republic of Korea; 2 HAE Research and Development Center, LG Electronics, Seoul, Republic of Korea; Ecole des Mines d'Alès, FRANCE

## Abstract

A culture-based colony counting method is the most widely used analytical technique for monitoring bioaerosols in both indoor and outdoor environments. However, this method requires several days for colony formation. In this study, our goal was fast monitoring (Sampling: 3 min, Detection: < 1 min) of indoor bioaerosol concentrations with ATP bioluminescence assay using a bioaerosol sampler. For this purpose, a novel hand-held electrostatic rod-type sampler (110 mm wide, 115 mm long, and 200 mm tall) was developed and used with a commercial luminometer, which employs the Adenosine triphosphate (ATP) bioluminescence method. The sampler consisted of a wire-rod type charger and a cylindrical collector, and was operated with an applied voltage of 4.5 kV and a sampling flow rate of 150.7 lpm. Its performance was tested using *Staphylococcus epidermidis* which was aerosolized with an atomizer. Bioaerosol concentrations were measured using ATP bioluminescence method with our sampler and compared with the culture-based method using Andersen cascade impactor under controlled laboratory conditions. Indoor bioaerosol concentrations were also measured using both methods in various indoor environments. A linear correlation was obtained between both methods in lab-tests and field-tests. Our proposed sampler with ATP bioluminescence method may be effective for fast monitoring of indoor bioaerosol concentrations.

## Introduction

Bioaerosol monitoring is useful for controlling air quality, assessing exposure in health risk evaluation studies, identifying emission sources, and estimating the performance of air cleaning devices. Exposure to bioaerosols can affect human health by causing infectious diseases, acute toxic reactions, and allergies [1–4]. A culture-based colony counting method is the most widely used analytical technique for monitoring bioaerosols in both indoor and outdoor environments [5–7]. However, this method requires several days for colony formation, which is one of its most serious limitations [8, 9]. In addition, the culture-based method is only applicable to culturable microbes that can divide at a sufficient rate to form colonies. Therefore, this method could underestimate the number of cells due to the presence of viable but non-culturable (VBNC) cells which can proliferate under certain conditions.

Previous studies have reported the feasibility of rapidly quantifying (or identifying) bioaerosols using airborne particle fluorescence spectrometry, pyrolysis-gas chromatography-ion mobility spectrometry (Py-GC-IMS), or bioaerosol mass spectrometry (BAMS) [10–14]. Instruments in which these techniques are used typically are either difficult to operate or expensive. With increasing concerns about biological contamination of indoor environments, a bioaerosol device that monitors indoor air quality needs to be developed for use in situ.

Adenosine triphosphate (ATP) bioluminescence is an available and affordable solution for rapidly monitoring bioaerosols in various environments. This assay uses ATP, which plays a central role as an intermediate carrier of chemical energy and links catabolism to biosynthesis within microbial cells. In the ATP assay, firefly luciferase catalyzes a reaction between luciferin and ATP, which causes luciferin to become excited and emit photons with a peak intensity in the 500 nm range as it returns to its ground energy level state [15]. Since the intensity of the light produced is directly proportional to the ATP content (which is proportional to biomass), it is possible to quantify the microbial biomass (the total amount of biological material derived from living, or recently living organisms) by measuring the ATP content using bioluminescence [16].

ATP assay-based methods have been employed to a limited extent for bioaerosol exposure monitoring in workplaces and confined environments [16]. Stewart et al. [17] used ATP-based assays for rapid enumeration of bioaerosols under controlled temperature and humidity conditions. Bioaerosols were sampled and dispersed into a sterile phosphate-magnesium buffer, and then measured using the ATP bioluminescence method. They were able to estimate bioaerosol numbers using this method within 2.5 h. Lee et al. [18] developed a biosensor to detect ATP from hydrosolized test bacteria using an aerosol condensation system, a microfluidic channel, and an ATP bioluminescence transducer. This sensor could determine the existence of bioaerosols within 10 min. Seshadri et al. [16] applied the ATP bioluminescence method to characterize the performance of bioaerosol sampling devices. The analytical time for this method was under 1 h. Yoon et al. [19] estimated the efficacies of air controlling devices in situ using ATP bioluminescence in combination with an inertial impactor. The bioaerosol concentration was estimated within 25 min using this method. Park et al. [20] used corona-generated air ions for cell-lysis to detect the ATP content of indoor bioaerosols. The total time required for sampling, cell-lysis, and bioluminescence detection was 40 min.

Swab-based commercial luminometer has been used to detect microbial contamination of solid and liquid specimens using ATP bioluminescence. In S1 Information, details of the swab-based luminometer are stated. In order to use this commercial luminometer to measure airborne microbial contamination, aerosol sampling is needed. Among various aerosol sampling technologies, the use of electrostatic sampling is increasing [21]. In an electrostatic sampler, airborne particles are electrically charged and then removed from the air stream by an electrical field. Sampling of airborne particles by electrostatic samplers has been widely studied from both theoretical and practical points of view, owing to its widespread practical applications. Mainelis et al. [22] suggested an electrostatic precipitator for bioaerosol collection. In their device, incoming biological particles are charged by two ionizers and then deposited onto a growth medium (agar) by an electrical field. Han et al. [23, 24] designed their electrostatic sampler with a super hydrophobic collection channel and half cylinder-shaped ground electrodes to collect bioaerosols into small amounts of liquids. They adapted the ATP bioluminescence method in order to analyze collection efficiency and the concentration rates of the sampler [25]. Miller et al. [26] developed a hand-held electrostatic precipitator for bioaerosol sampling. They designed a point-to-plane electrostatic precipitator particle sampler that used a TEM grid or metallic foil substrate as the collecting plate. Tan et al. [21] designed an automated electrostatic sampler with the capability to integrate with other real-time biosensor devices. The sampler was designed using a half-ball shaped steel electrode. Roux et al. [27] suggested a single stage electrostatic sampler for the efficient collection of microorganisms at a flow rate of 100 lpm.

In this study, our goal was fast monitoring of indoor bioaerosol concentrations with ATP bioluminescence assay using a bioaerosol sampler. A novel electrostatic sampler was designed to be easily combined with the commercial swab-based type luminometer. The sampler was field portable (pump-less, small, and light) and had fast sampling capabilities. Laboratory and field tests in various indoor environments were carried out using our sampler.

## Materials and Method

### Preparation of test bacterial suspension

In this study, *Staphylococcus epidermidis* was used for the performance test. ISO 14698–1 suggests that *S*. *epidermidis* is a suitable strain for testing the biological efficiency of air samplers used for counting airborne microbes. A bacterial suspension of *S*. *epidermidis* (ATCC 14990) was prepared by culturing 0.1 mL of an overnight culture inoculated in 15 mL of a nutrient broth for 24 h at 37°C. The nutrient broth was prepared by dissolving 5 g of peptone and 3 g of meat extract in 1000 mL of sterile deionized water. The solution was sterilized in an autoclave. For bioaerosol generation, the prepared bacterial suspension was washed three times with sterile deionized water using a centrifuge (VS-1500N, Vision Scientific, Korea) at 6000 rpm (2697g) for 15 min to remove the residual particles, including the components of the nutrient broth.

### Design of the sampler

**Fig 1:**
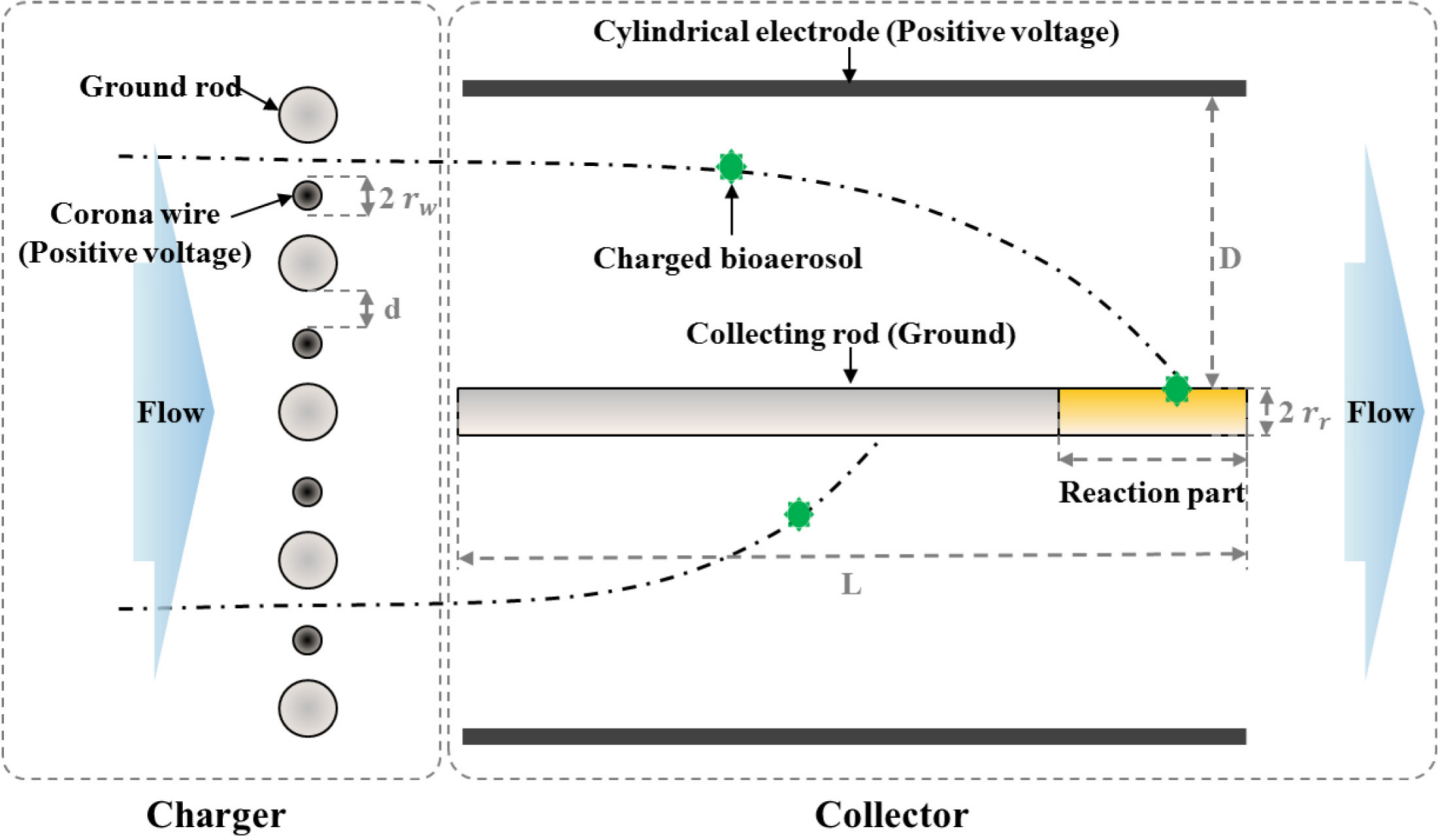
Schematic of the sampler.

The schematic of our electrostatic bioaerosol sampler is shown in Fig 1. The sampler consists of a wire-rod corona charger and a cylindrical collector. During sampling, air enters the collector through the wire-rod charger causing particles in the air to become positively charged. Next, the charged particles are collected on the collecting rod by an externally applied electric field. After sampling, the collecting rod is detached from the sampler and placed into a commercial swab holder (Lucipac W, Kikkoman, Japan). Therefore, the rod is used as a substitute of the swab stick used in a commercial swab-based type luminometer. Finally, the swab holder is placed into the measuring chamber of the ATP measuring device, where the relative light unit (RLU) value is displayed. In S2 Information, our design process of the sampler is stated in detail.

The charger consisted of four discharge wires (tungsten) and five ground rods (stainless steel) in parallel. In this study, the radii of the discharge wire (*r*_*w*_), the radii of the ground rod (*r*_*r*_), and the distance between the wire and the rod (*d*) were 20 μm, 2 mm, and 3.98 mm, respectively. The collector consisted of a collecting rod (stainless steel) and a cylindrical electrode (stainless steel). The collecting rod was designed to fit into a commercial swab holder. The radius and the length of the collecting rod were 2 mm and 100 mm, respectively. The distance between the collecting rod and the cylinder electrode was 17 mm.

For fast monitoring of bioaerosol concentration with the ATP bioluminescence assay, bioaerosols should be concentrated by the sampler in a short time. The areal density of bioaerosols (*ρ*_*s*_, #/*m* ^2^) deposited on the collecting rod is expressed by the following equation

$$\rho_{s}\;=\frac{NQ\eta_{c}t}{A_{c}}$$ (1)

where *N* is the concentration of bioaerosols in the air, *Q* is the flow rate of the sampler, *η*_*c*_ is the collection efficiency, *t* is the sampling time, and *A*_*c*_ is the surface area of the collecting rod. In order to increase the sampled cell density under constant sampling time and bioaerosol concentration, the deposition velocity (*V*_*c*_) defined below,

$$V_{c}=\frac{N_{c}}{N\cdot t}$$ (2)

should be increased. Therefore, a higher sampling flow rate and/or a higher collection efficiency results in a higher deposition velocity.

### Performance test of the sampler

**Fig 2:**
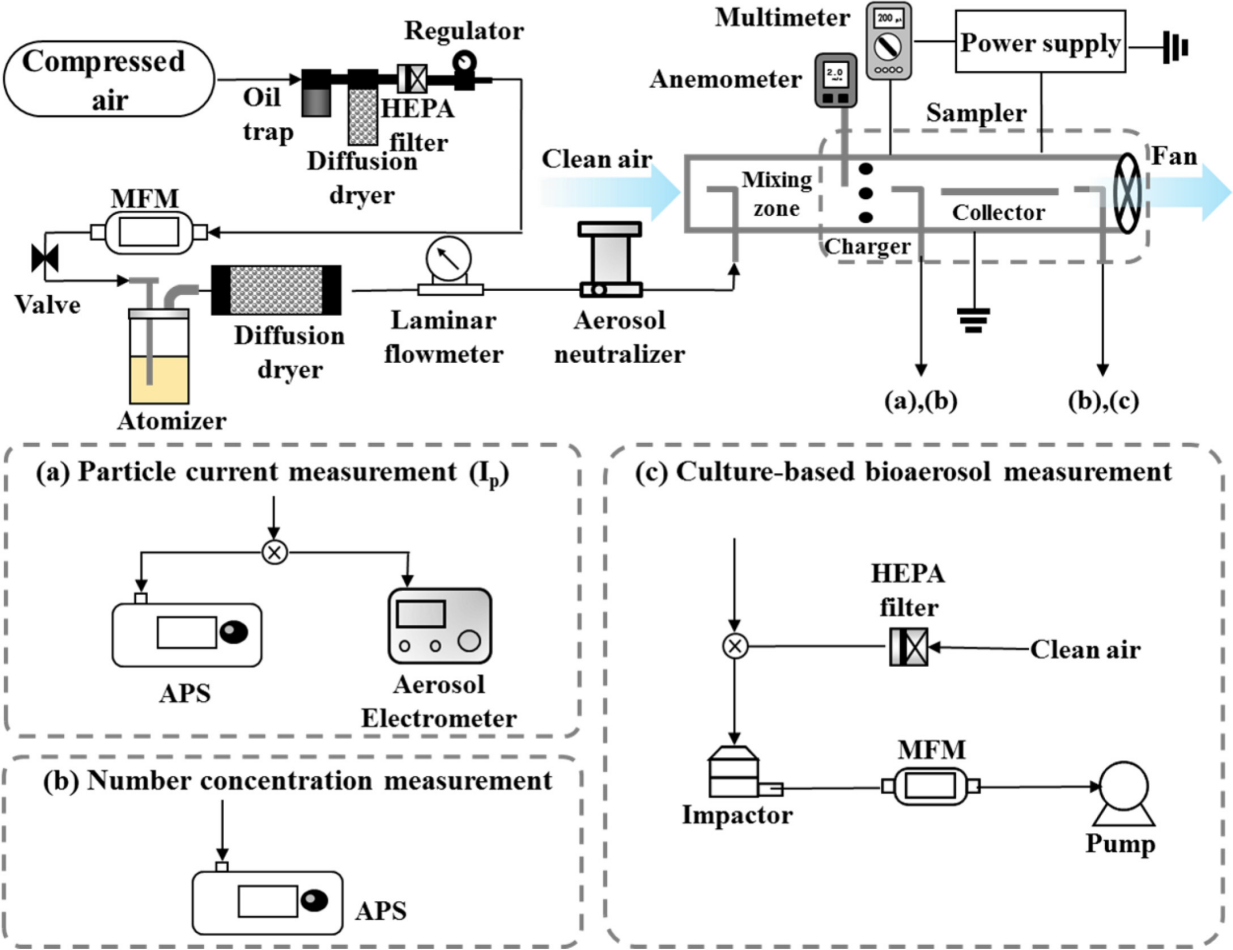
Schematic diagram of the experimental setup for the performance tests of the sampler.

Aerosolized *S*. *epidermidis* was used for performance test of the sampler. Performance tests of the sampler were carried out in a clean booth (Clean booth, Kumkang, Korea). Experimental setup of the performance tests are shown in Fig 2. The bacterial suspension of *S*. *epidermidis* was aerosolized using a Collison-type atomizer (9302, TSI Inc., USA). Compressed air from a dry-cleaned air supply system consisting of an oil trap, diffusion dryer, and high efficiency particulate air (HEPA) filter was delivered to the atomizer. Dry-cleaned air at a flow rate of 3 lpm formed a high-velocity jet through an orifice in the atomizer. The flow rate was controlled by a valve and was measured by a mass flowmeter (MFM; model 4143, TSI Inc., USA). The pressure drop from the jet drew the bacteria solution up through a tube. The solution was then broken up into droplets by a high velocity air jet. The resultant larger droplets impinged on an impactor while the smaller droplets made no contact and formed an aerosol that exited through an outlet. The bioaerosols from the atomizer were passed through a diffusion dryer to remove humidity. The flow rate of air containing the bioaerosols was measured by a laminar flowmeter. After charge neutralization by a neutralizer (Soft X-ray charger 4530, HTC Co. Ltd., Korea), aerosolized *S*. *epidermidis* was diluted by clean air in the mixing zone of a test duct. The flow rate of the clean air was controlled by a fan attached at the end of the test duct and was measured using an anemometer (Veloci Check, TSI Inc., USA) in front of the charger. The diluted bioaerosols were then charged by the charger (dilution ratio = 12.5–50). For total flow rates between 56.5 and 150.7 lpm, the number concentrations after mixing with clean air were between 35 and 2910 #/cm^3^.

Fig 2A shows the schematic diagram of the experimental set up for measuring the number of charges of a bioaerosol particle. At the exit of the charger, the bioaerosols were sampled by both an aerodynamic particle sizer (APS; model 3321, TSI Inc., USA) and an aerosol electrometer (model 3068B, TSI Inc., USA). The APS measures concentrations of particles with an aerodynamic diameter between 0.5 and 20 μm using a double-crest optical system. The sampling flow rate was 5 lpm. The aerosol electrometer measures the total electrical charge carried by the bioaerosols and gaseous ions. The sampling flow rate of the electrometer was also 5 lpm. The average charge of a bioaerosol particle is defined as

$$n_{avg}=\frac{I-I_{i}}{N_{c}eQ}$$ (3)

where *I* is the electrical current of the charged particles and gaseous ions, *I*_*i*_ is the electrical current of the ions, *N*_*c*_ is the number concentration of the charged particles exiting the charger, and *e* is the charge of an electron.

The wall loss (*L*) of the charged bioaerosols in the charger was tested with the schematic shown in Fig 2B. The penetration ratio is defined as

$$P=1-L=\frac{N_{charger,on}}{N_{charger,off}}$$ (4)

where *N*_*charger*,*off*_ is the number concentration of bioaerosols when the power supplied to the charger is turned off, and *N*_*charger*,*on*_ is the number concentration of bioaerosols escaping from the charger when the power is being supplied to the charger (= *N*_*c*_). These number concentrations were measured at the exit of the charger.

To determine the collection efficiency, bioaerosols were generated and charged with the same procedure as used in the charging test. At the exit of the collector, the bioaerosols were sampled by the APS (see Fig 2B). The collection efficiency can be defined experimentally as follows:

$$\eta_{c}=1-\frac{N_{sampler,on}}{N_{sampler,off}}\times \frac{1}{P}$$ (5)

where *N*_*sampler*,*off*_ is the number concentration of bioaerosols when the power supplied to the charger and the collector are turned off (= *N*), and *N*_*sampler*,*on*_ is the number concentration of charged bioaerosols escaping from the sampler when power is being supplied to the charger and the collector.

### Laboratory test of bioaerosol detection

Laboratory tests of ATP bioluminescence assay with the sampler were carried out with *S*. *epidermidis* bioaerosols. Bioaerosols were sampled on the collecting rod for 10 s. After sampling, the collecting rod was put into the commercial swab holder, and then the sampled bioaerosols were mixed with the ATP-releasing reagent and the luminescent reagent. ATPs were extracted from the bioaerosols and light was emitted. The swab holder with the collecting rod was placed directly into the luminometer (Lumitestor, Kikkoman, Japan), and the light intensity was measured to provide an RLU value.

The culture-based method was also performed to compare with the bioluminescence detection method. A schematic of the experimental setup for the culture-based method is shown in Fig 2C. A sixth stage of Andersen cascade impactor (TE-10-800, Tisch Environmental, USA) with a cutoff diameter of 650 nm was used for this purpose. The impactor required a sampling flow rate of 28.3 lpm. 1 lpm of air including bioaerosols entered the impactor through the sampler. The remaining 27.3 lpm of required flow was provided by clean air through a HEPA filter. The bioaerosols were sampled on a nutrient agar plate in the impactor for 10 s and then cultured in the incubator during 7 days at 30°C. After incubation, the colony forming units (CFU) were counted. Since colony superposition occurs when microbial particles impact the same spot through the same sieve hole, it is necessary to revise the enumerated colony data with the positive hole correction formula [28].

### Field test of indoor bioaerosol detection

**Fig 3:**
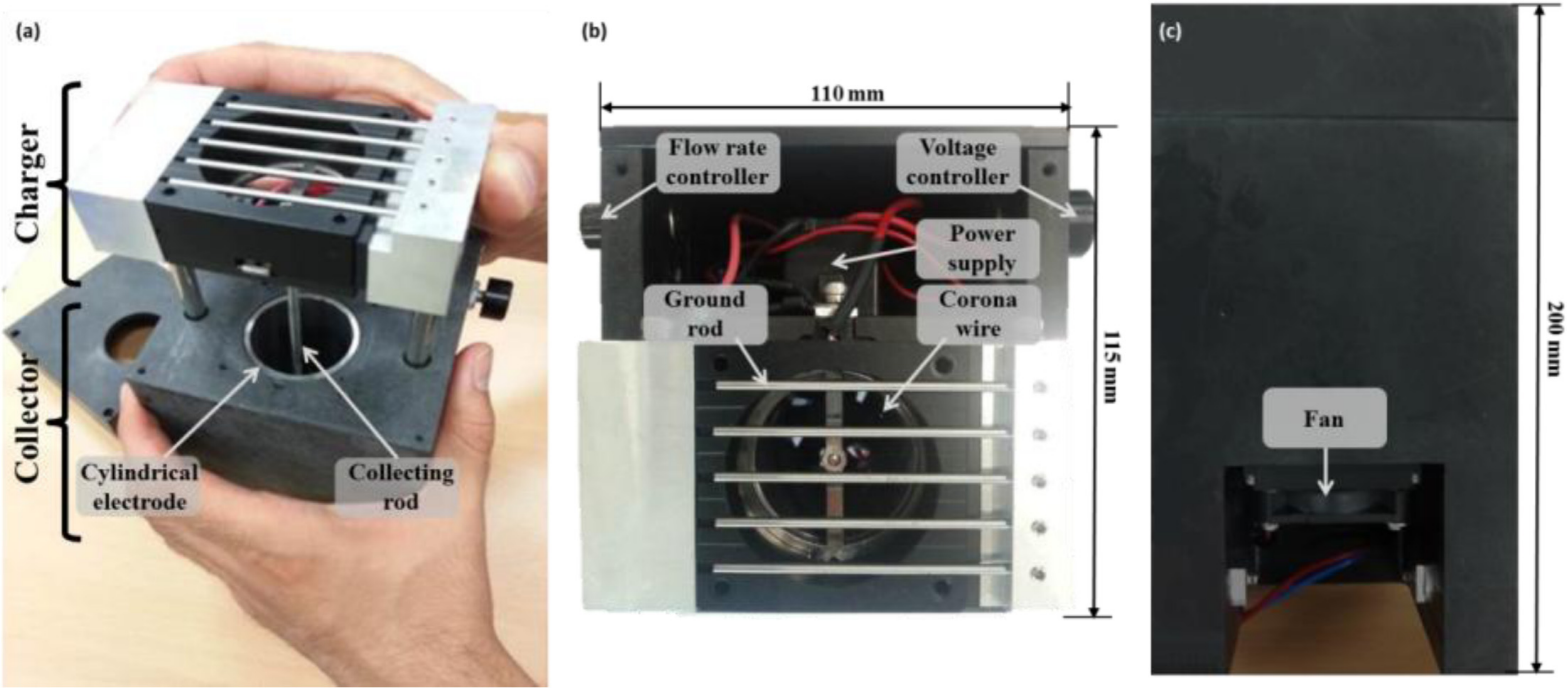
The prototype of our electrostatic sampler for bioaerosol detection; (a) Front view, (b) Top view, and (c) Side view.

After laboratory tests, a prototype hand-held electrostatic sampler was fabricated for field tests. Fig 3 shows photos of our electrostatic sampler, which consists of a charger, a collector, a fan (VD5010B12M, Trohito, China), a power supply (G50, EMCO, USA) and two lab-made controllers. The dimensions of the prototype are 110 × 115 × 200 mm in width, length, and height, respectively.

The prototype sampler was applied to detect indoor bioaerosols at various locations including an occupational office, a cafeteria in a university, a dining room in a kindergarten, a classroom in a kindergarten, and an auditorium located in Seoul, Korea. The field test at each location was carried out on five different days. Each measurement was repeated three times. Temperature and relative humidity were 19.5–26.8°C and 22–43%, respectively. The sampler was located at a height of about 1 m in the middle of the test space. At the same time and location, the culture-based counting method using Andersen cascade impactor was carried out for comparison. A nutrient agar was used for a culture medium in the field test. The sampling time for both methods was 3 min.

### Ethics Statement

The bioaerosol sampling was carried out at public places of our university. Therefore, specific permission was not required. The field studies did not involve endangered or protected species.

## Results and Discussion

### Evaluation of the sampler

The corona discharge of the charger was generated from each discharge wire, resulting in the formation of positive ions, which moved along the electric field to a grounded rod. As the applied voltage of the charger increased beyond the corona starting voltage of 3kV, the corona current gradually increased. The spark was triggered for applied voltages above 5 kV. The corona currents for various applied voltages were measured and the results are shown in Fig Ba in S3 Information. In consideration of a stable operation, 4.5 kV was selected for the applied voltage of the charger. The average charges of *S*. *epidermidis* bioaerosols for different flow rates at an applied voltage of 4.5 kV are shown in Fig Bb in S3 Information. The average number of charges per particle decreased from 530 to 465 as the flow rate increased from 56.6 to 150.7 lpm. The penetration ratios (*P*) of the charger for different flow velocities and flow rates at an applied voltage of 4.5 kV are in Fig Bc in S3 Information. When the total flow rate was changed from 56.5 to 150.7 lpm, the penetration ratio was changed from 70% to 90%. Details of the results are stated in S3 Information.

**Fig 4:**
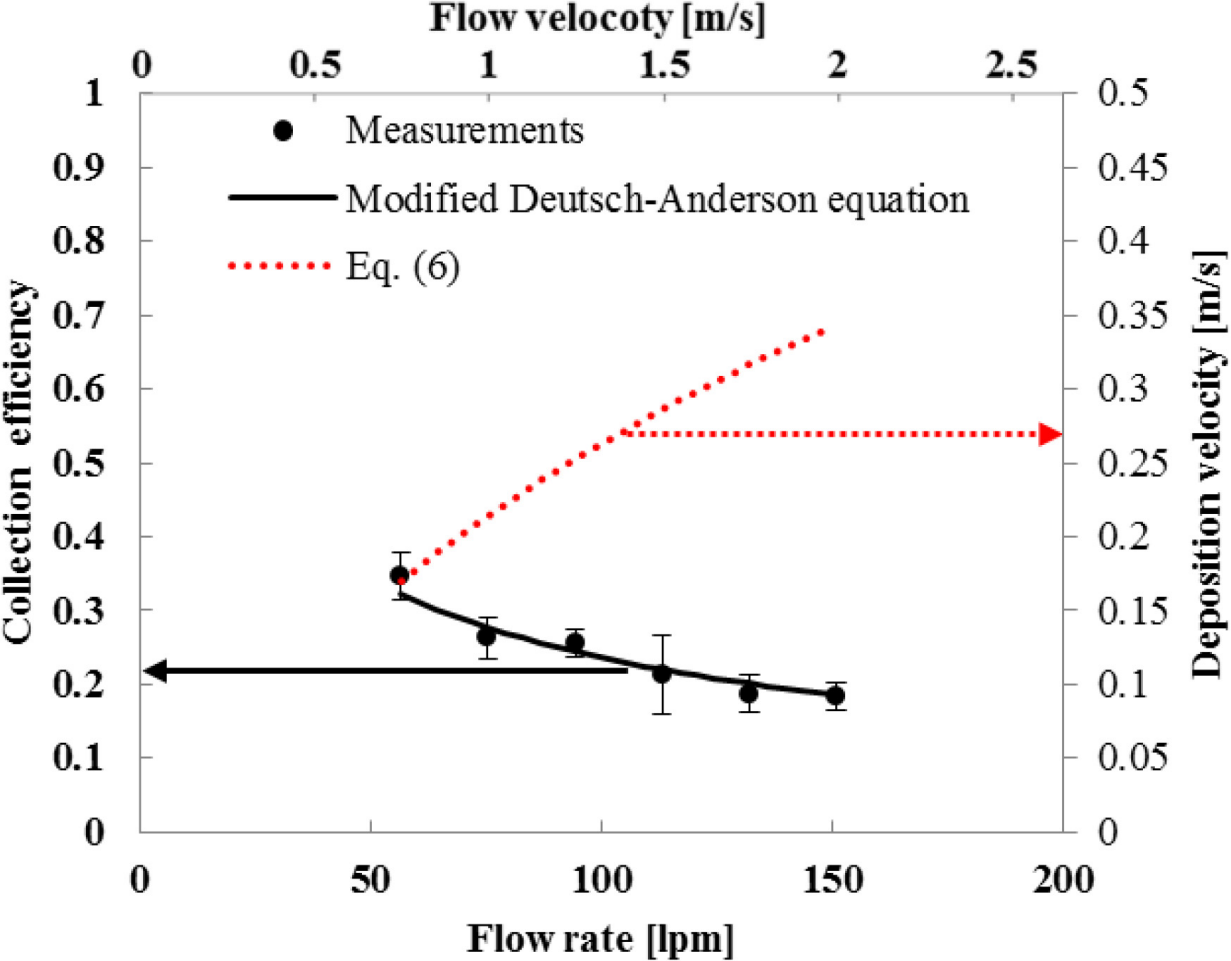
Collection efficiencies and deposition velocities for various flow rates (S. epidermidis, Applied voltage at the charger and the collector is 4.5 kV)

In this work, the applied voltage of the collector was chosen to be the same as that of the charger (4.5 kV), in order to simplify the design of a power pack. Fig 4 shows experimental results of collection efficiency and deposition velocity of *S*. *epidermidis* bioaerosols at different flow rates. The experimental results of the collection efficiency were in good agreement with the modified Deutsch-Anderson equation (See Eq. A in S2 Information) which was used for design of the collector. Fig 4 also shows the relationship between flow rate and deposition velocity. Considering the penetration ratio (*P*) of the charger, the equation for deposition velocity Eq (2) was modified as follows:

$$V_{c}=\frac{Q}{A_{c}}\times \eta_{c}\times P$$ (6)

As the flow rate increased, the collection efficiency decreased and the deposition velocity increased. Since the deposition velocity represents the number of bacteria captured in the collecting rod per unit time for a given test bacteria concentration, its maximum value of 0.336 m/s (corresponding to the maximum flow rate of 150.7 lpm) was chosen for this study. At this condition, the penetration ratio of the charger was 0.904 ± 0.050.

### Laboratory test of bioaerosol detection

**Fig 5:**
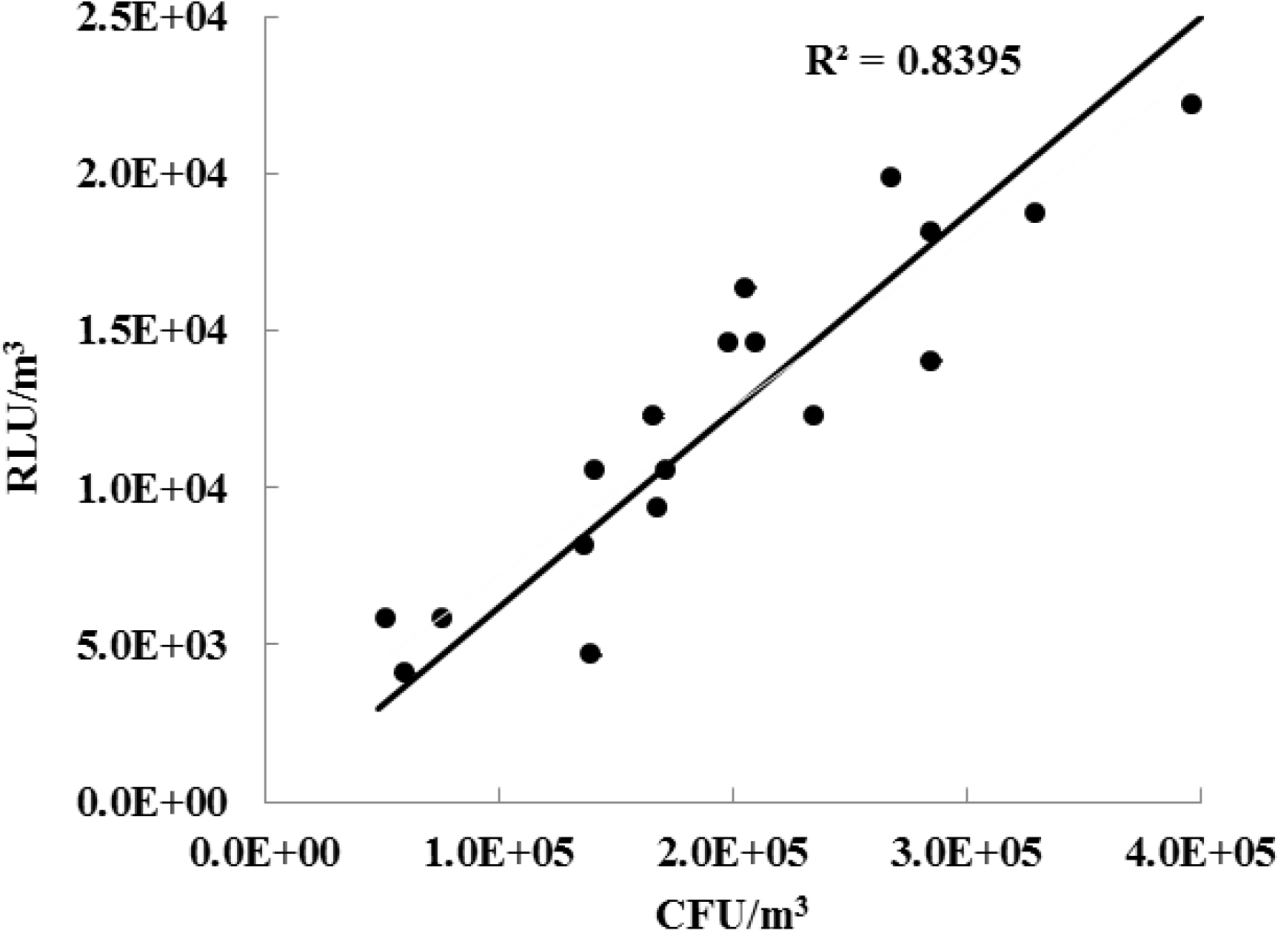
Correlation between RLU/m^3^ and CFU/m^3^ of *S*. *epidermidis*.

Fig 5 shows the correlation between the RLU/m^3^ of *S*. *epidermidis* bioaerosols measured using the ATP-based method with our sampler and the CFU/m^3^ obtained by the culture-based method using Andersen cascade impactor. The sampling flow rate, sampling time, and applied voltage to the sampler were chosen to be 150.7 lpm, 30 s, and 4.5 kV respectively. The unit conversion from CFU to CFU/m^3^ was made as follows:

$$\left[\text{CFU}/m^{3}\right]=\frac{\left[\text{CFU}\right]}{Q_{i}t_{i}\eta_{i}}$$ (7)

where *Q*_*i*_ is the sampling flow rate of the impactor, *t*_*i*_ is the sampling time of the impactor, and *η*_*i*_ the collecting efficiency of the impactor. The collecting efficiency of the impactor was 90.2% for 800 nm-sized particles. The experimental setup was stated in S4 Information.

Similarly, the unit conversion from RLU to RLU/m^3^ was made as follows,

$$\left[\text{RLU}/m^{3}\right]=\frac{\left[\text{RLU}\right]}{PQt\eta_{c}R_{r}}$$ (8)

where *t* is the sampling time of our sampler, *R*_*r*_ is the ratio between the length of the reaction part and the length of collecting rod (see Fig 1). For our setup, *R*_*r*_ was 0.4. In our study, all bioaerosols collected on the reaction part of the collecting rod were assumed to react with reagents in the swab holder. The concentrations measured using the culture-based method were (1.63 ~ 3.70) × 10^6^ CFU/m^3^, while the concentrations using the ATP-based method with the sampler were (1.02 ~ 2.12) × 10^5^ RLU/m^3^. Therefore, 1 RLU corresponded to 16.0 ± 4.4 CFU of aerosolized *S*. *epidermidis* cells. In our previous study [19], 1 RLU corresponded to 27.5 ± 8.7 CFU of aerosolized *S*. *epidermidis* cells. This discrepancy might be due to difference in the post-treatment methods used after sampling between the two experiments. Yoon et al. [19] sampled bioaerosols using an impactor and then manually swabbed the sampled area in the impactor using a swab stick. This is the suggested method when using a commercial swab-based type luminometer (see S1 Information). However, in this study, sampled bioaerosols on the rod were directly exposed to bioluminescence reagents. Therefore, we believe that more ATPs were extracted and reacted in this study compared to the study by Yoon et al. [19].

### Field test of indoor bioaerosol detection

**Fig 6:**
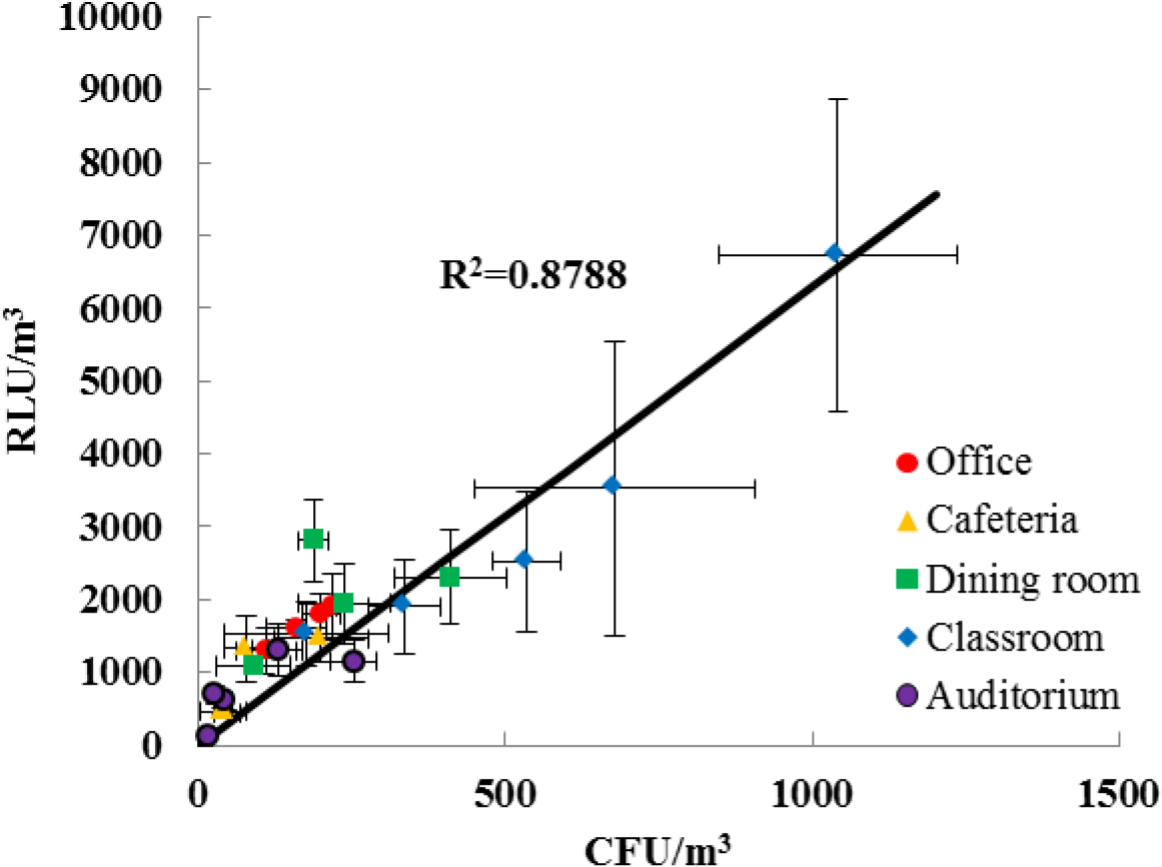
Correlation between the concentrations of indoor bioaerosol measured using ATP (RLU/m^3^) and culture-based (CFU/m^3^) methods in an occupational office, a cafeteria in a university, a dining room in a kindergarten, a classroom in a kindergarten, and an auditorium.

Fig 6 shows the correlation between the RLU/m^3^ of indoor bioaerosols measured using the ATP-based method with our sampler and the CFU/m^3^ measured by the culture-based method with the sixth stage of Andersen cascade impactor. Each data point represents an average value of RLU/m^3^ as well as CFU/m^3^ in a specific place and on a specific day. When the occupants in an indoor environment were more active, the bioaerosol concentrations and measurement errors (standard deviation values of RLU/m^3^ and CFU/m^3^) were higher in both in the culture-based method and the ATP method.

In the results of our field tests, 1 RLU corresponded to 0.16 ± 0.05 CFU of indoor bioaerosols; this value was very different compared to the correlation obtained in the laboratory test. We believe this difference is due to the presence of fungal spores and VBNC bioaerosols [19, 20]. Previous studies on ATP methods reported that different bioaerosols had varying levels of ATP [16–18]. For example, fungi generally contain more ATP than bacteria. In a study carried out by Thore et al. [29] with *E*. *coli*, *Staphylococcus* spp., *Proteus* spp., *Streptococcus* spp., *Klebsiella* spp., *Pseudomonas* spp., mixed infections, and unidentified bacteria, the average ATP level per bacterial cell was reported to be 3.6 × 10^–18^ mol. Rakotonirainy et al. [30] stated that the ATP level per fungal spore (*Aspergillus niger*, *A*. *fumigatus*, *A*. *versicolor*, *A*. *flavus*, *Penicillium chrysogenum*, *Eurotium chevalieri*, *Neosartorya fischeri*, *Chaetomium globosum*, and *Ulocladium* spp.) was 3.3 × 10^–17^ mol. According to previous study of VBNC cells, most bioaerosols are nonculturable [31, 32]. Bioaerosols in the VBNC state fail to grow on the nutrient media that is normally used to grow and develop colonies. However, these bioaerosols are alive and capable of renewed metabolic activity. ATP levels, which decline rapidly in dead cells, remain high in VBNC cells [33]. Therefore, they could significantly contribute to the RLU value but not to the CFU value.

Different indoor environments may contain various microorganisms with different ATP contents. However, it was interesting to note that the correlation (= RLU/CFU) did not differ significantly in the different indoor environments that we tested (see Fig 6). The reason for this might be related to typical strains of indoor bioaerosols. Mandal and Brandl [34] reviewed and summarized forty previous published studies carried out in field tests for indoor environments. They reported that the genera *Bacillus*, *Micrococcus*, *Kocuria* and *Staphylococcus* are representatives of typical bacterial strains found in indoor atmosphere. They also reported that fungal strains which are proportionally of importance in indoor air samples are comprised of the genera *Alternaria*, *Aspergillus*, *Cladosporium*, and *Penicillium*.

## Conclusions

A field portable bioaerosol sampler (110 mm wide, 115 mm long, and 200 mm tall) was developed for use in fast monitoring (Sampling: 3 min, Detection < 1 min) of indoor bioaerosols concentration. For this purpose, a novel hand-held electrostatic rod-type sampler was developed and used with a commercial luminometer, which uses the ATP bioluminescence method. The sampler consisted of a wire-rod type charger and a cylindrical collector, and was operated with an applied voltage of 4.5 kV and a sampling flow rate of 150.7 lpm. A linear correlation of RLU/CFU ≅ 6.3 was obtained between our method and the culture-based method in the field tests, even though the measurements were carried out in various indoor environments. Our proposed sampler with ATP bioluminescence method may be effective for fast monitoring of indoor bioaerosol concentrations.

## Supporting Information

S1 Information Test procedure using a commercial swab-based type luminometer. (DOCX)

S2 Information Design of the sampler. (DOCX)

S3 Information Evaluation of the sampler. (DOCX)

S4 Information Laboratory test of bioaerosol detection. (DOCX)
