# Supplementary material for: Fast Monitoring of Indoor Bioaerosol Concentrations with ATP Bioluminescence Assay Using an Electrostatic Rod-Type Sampler
Source: PLoS One. 2015 May 7;10(5):e0125251. doi: 10.1371/journal.pone.0125251 (PMC4423956; doi:10.1371/journal.pone.0125251)
Supplement: S1 Information — (DOCX) [file pone.0125251.s001.docx]

**Test procedure using a commercial swab-based type luminometer**


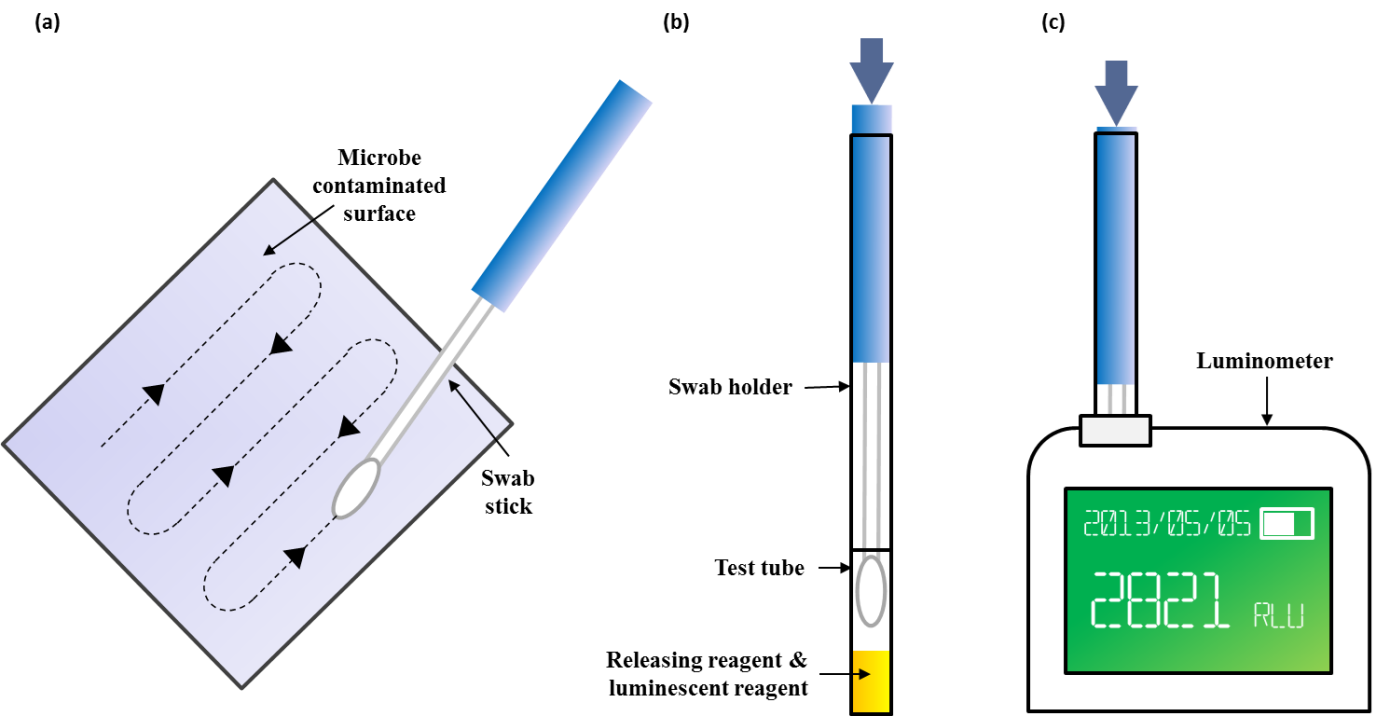


**Fig. A. Test procedure using a commercial swab-based type luminometer.**

With a swab-based type, the inspection area is swabbed (Fig. Aa), and then the swab stick is placed into the swab holder (Fig. Ab) containing both the ATP-releasing reagent and the luminescent reagent. The ATP-releasing reagent extracts ATPs from the sampled cells into a test tube, and the luminescent reagent reacts with the ATPs to generate light in the presence of oxygen. When the swab holder is placed directly into the measurement chamber of the luminometer, ATP bioluminescence values are expressed in relative luminescent units (RLUs) (Fig. Ac) [1].

[1] Hawronskyj JM, Holah J (1997) ATP: A universal hygiene monitor. Trends Food Sci. Tech. 8: 79-84.
