# Supplementary material for: Fast Monitoring of Indoor Bioaerosol Concentrations with ATP Bioluminescence Assay Using an Electrostatic Rod-Type Sampler
Source: PLoS One. 2015 May 7;10(5):e0125251. doi: 10.1371/journal.pone.0125251 (PMC4423956; doi:10.1371/journal.pone.0125251)
Supplement: S2 Information — (DOCX) [file pone.0125251.s002.docx]

**Design of the sampler**

**Design of the collector**

The modified Deutsch-Anderson equation of Bai et al. [1] was used to determine the collection efficiency ($\eta_{c})$,

$\eta_{c}=1-\exp\left( -{N_{De}}^{c} \right)$ (A)

where $N_{De}$ is the Deutsch number defined as:$N_{De}=\frac{wA_{s}}{Q}$ (B)

where $c$ is a constant ranging from 0.4 to 0.6, $w$ is the electrical migration velocity of a particle, $A_{s}$ is the surface area of the sampler ($2\pi DL$, where $L$ is the length of the collecting rod and, $D$ is the distance between the cylinder and the collecting rod) and $Q$ is the sampling flow rate. The electrical migration velocity of a particle, $w$, is calculated using the following equation

$w=\frac{n_{p}eC_{c}E}{3\pi\mu d_{p}}$ (C) where $n_{p}$ is the number of particle charges, $e$ is the charge of an electron, $E$ is the electric field intensity, $\mu$ is the dynamic viscosity of the air, and $d_{p}$ is the particle diameter. $C_{c}$ is the slip correction factor [2]:

$C_{c}=1+\frac{\lambda}{d_{p}}\left[ 2.34+1.05exp(-0.39\frac{d_{p}}{\lambda}) \right]$ (D)

where $\lambda$ is the mean free path of air which is 0.066 μm at 1 atm and 293 K [3].

**Design of the charger**

It is necessary to increase the particle charge ($n_{p})$in order to increase the collection efficiency ($\eta_{c}$). The number of particle charges is estimated using both diffusion and field charging theories [3]:

$n_{p}=n_{d}+n_{f}$ (E)

$n_{d}=\frac{d_{p}kT}{2K_{E}e^{2}}ln\left[ 1+\frac{\pi K_{E}d_{p}c_{i}e^{2}N_{i}t_{c}}{2kT} \right]$ (F)

$n_{f}=\left( \frac{3\varepsilon_{p}}{\varepsilon_{p}+2} \right)\left( \frac{Ed_{p}^{2}}{4K_{E}e} \right)\left( \frac{\pi K_{E}eZ_{i}N_{i}t_{c}}{1+\pi K_{E}eZ_{i}N_{i}t_{c}} \right)$ (G)

where $n_{d}$ is the number of charges acquired by a particle via diffusion charging, $n_{f}$ is the number of charges acquired by a particle via field charging, $k$ is the Boltzmann constant,$T$ is the absolute temperature,$K_{E}$ is the constant of proportionality $\frac{1}{4\pi\varepsilon_{0}}$ (where $\varepsilon_{0}$is the permittivity of a vacuum), $Z_{i}$ is the ion mobility, $c_{i}$ is the mean velocity of air ions ($240 m/s)$, $N_{i}$ is the ion number concentration, *t* is the residence time in the charger, and $\varepsilon_{p}$ is the relative permittivity of the particle.

In order to obtain a higher particle charge, a higher ion concentration is required. The ion concentration under corona discharge is expressed as:

$N_{i}=\frac{I_{corona}}{eZ_{i}EA_{e}}$ (H)

where $I_{corona}$ is the corona current and $A_{e}$ is the surface area of the electrode. The corona current can be expressed using the general empirical formula for the current-voltage relation reported by Meng et al. [4],

$I_{corona}=C{(\phi-\phi_{0})}^{\alpha}$ (I) where $C$ and $\alpha$ are constants depending on the electrode geometry, $\phi$ is the applied voltage on the discharge wire, and $\phi_{0}$ is the corona starting voltage. The exponent $\alpha$ is between 1.5 and 2.0. When the applied voltage exceeds the breakdown voltage, the electric field intensity exceeds the dielectric field strength of air (3 MV/m) and a spark is triggered.

To produce a corona discharge, it is essential that the electric field strength is beyond the corona starting electric field. When two distant, parallel, and unequal cylindrical conductors are used as electrodes, the corona starting voltage is calculated using the following equation

$\phi_{0}=2E_{0}r_{w}ln \left[ \frac{d}{\sqrt{r_{w}r_{r}}} \right] for d\gg r_{r}$ (J) where $r_{w}$ and $r_{r}$ are the radii of the discharge wire and the ground rod, respectively, and $d$ is the distance between the wire and the rod [5]. The corona starting electric field,$E_{0}$, is defined as

$E_{0}=f\times3.1\times{10}^{6}(\delta+0.0308\left( \delta/r_{w} \right)^{0.5})$ (K)

where $f$ is a factor that accounts for wire roughness and $\delta$ is the correction coefficient of temperature and pressure.

**References**

[1] Bai H, Lu C, Chang CL (1995) A model to predict the system performance of an electrostatic precipitator for collecting polydisperse particles. J. Air Waste Manage. 45 (11): 908-916.

[2] Allen MD, Raabe OG (1982) Re-Evaluation of Millikan’s Oil Drop Data for the Motion of Small Particles in Air. J. Aerosol Sci. 6: 537-547.

[3] Hinds WC (1999) Aerosol Technology: Properties, Behavior, and Measurement of Airborne Particles, second ed. Wiley.

[4] Meng X, Zhang H, Zhu J (2008) A general empirical formula of current-voltage characteristics for point-to-plan geometry corona discharges. J. Phys. D. Appl. Phys. 41: 065209.

[5] Kaiser KL (2006) Electrostatic discharge. CRC press.
