# Supplementary material for: Fast Monitoring of Indoor Bioaerosol Concentrations with ATP Bioluminescence Assay Using an Electrostatic Rod-Type Sampler
Source: PLoS One. 2015 May 7;10(5):e0125251. doi: 10.1371/journal.pone.0125251 (PMC4423956; doi:10.1371/journal.pone.0125251)
Supplement: S3 Information — (DOCX) [file pone.0125251.s003.docx]

**Evaluation of the sampler**

The corona currents for various applied voltages were measured and the results are shown in Fig. Ba. As the applied voltage increased beyond the corona starting voltage of 3kV, the corona current gradually increased. The spark was triggered for applied voltages above 5 kV. The corona starting voltage that was theoretically predicted by Eq. J was 3.2 kV: this value is close to the measured one. The experimental data of the current-voltage characteristics fitted the theoretical values determined with Eq. I when $C$ = 3.6 $\times$ 10^-3^ A/V^1.5^ and $\alpha$ = 1.5 were used. The ion concentration ($N_{i})$ was then estimated using Eq. 8. We assumed that $Z_{i}$ was $1.4 \times{10}^{-4} m^{2}/V\cdot s$ for positive air ions [1]. The ion concentrations were between 5.58 $\times$ 10^15^ and 1.23 $\times$ 10^16^ #/m^3^ for applied voltages between 4 and 5 kV.

After the ion concentrations were estimated, the average particle charge numbers were calculated using Eqs. E – G. For this purpose, the APS data were used. The geometric mean and geometric surface-mean diameter of the bioaerosols were 835 and 923 nm, respectively. The geometric standard deviation of the size distribution was 1.24. Ji et al. [2] used scanning electron microscopy (SEM) to show *S. epidermidis* is spherical with a geometric mean diameter of ~ 800 nm. Since a bacterial cell is composed of about 70 % water (with the other 30 % made up of structural and functional molecules), we assumed in our calculation that a bioaerosol particle is similar to a water particle. Average particle charges were also obtained from current measurements with Eq. C of the text. Fig. Bb shows the average charges of *S. epidermidis* bioaerosols for different flow rates at an applied voltage of 4.5 kV. When the flow rate was increased, the particle charge decreased due to the decrease of particle residence time in the charger. The calculated particle charges were similar to the experimental data.

Fig. Bc shows the penetration ratios ($P$) of the charger for different flow velocities and flow rates at an applied voltage of 4.5 kV. The results were fitted to the following empirical equation:

$P=1-\beta_{1}\times exp \left( -\beta_{2}\frac{Q}{A} \right)$ (L) where the constants $\beta_{1}$ and $\beta_{2}$ are 0.6 (dimensionless) and 1.1 s/m, respectively, and $A$ is the cross-sectional area of the charger. When the sampling flow rates were low, the particles were highly charged but they may be more easily captured on the ground electrode of the charger. As the flow rate increased to 2 m/s, the penetration ratio increased to about 90%.


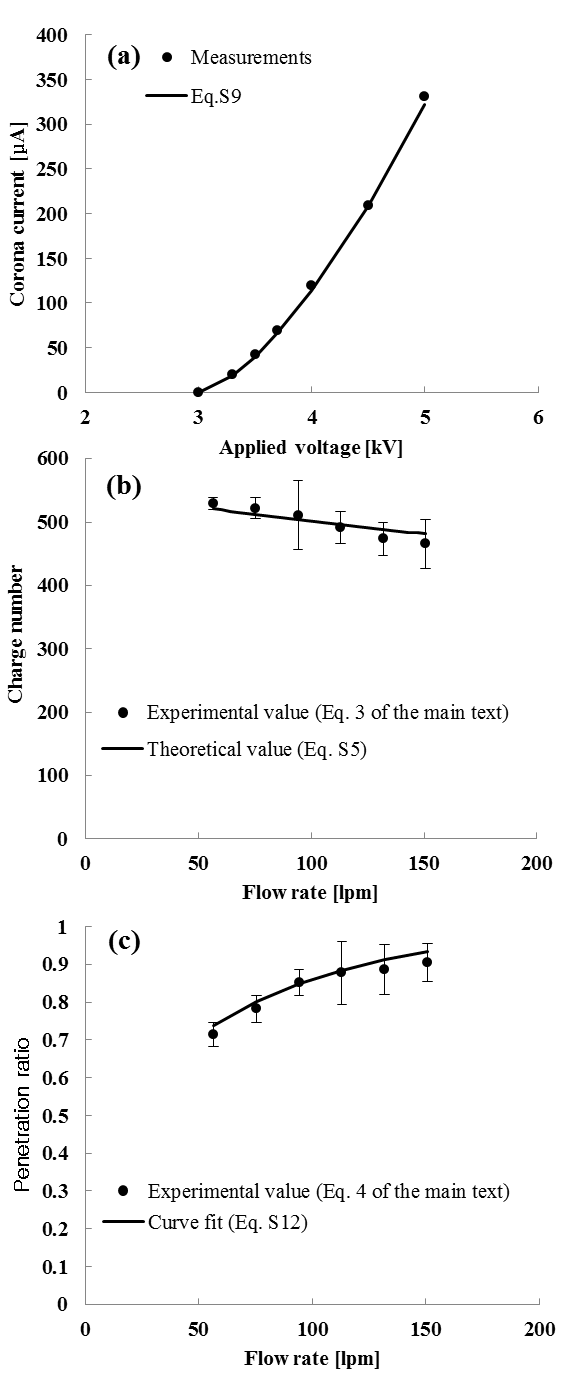


**Fig. B. Performance tests of the charger: (a) Electrical characteristics of the charger, (b) Effect of flow rate on particle charge (*S. epidermidis*, applied voltage at the charger is 4.5 kV), and (c) Effect of flow rate on penetration ratio of the charger (*S. epidermidis*, applied voltage at the charger is 4.5 kV).**

**References**

[1] Hinds WC (1999) Aerosol Technology: Properties, Behavior, and Measurement of Airborne Particles, second ed. Wiley.

[2] Ji JH, Bae GN, Yun SH, Jung JH, Noh HS, et al. (2007) Evaluation of a silver nanoparticle generator using a small ceramic heater for inactivation of *S. epidermidis* bioaerosols. Aerosol Sci. Tech. 41: 786-793.
