# Supplementary material for: Fast Monitoring of Indoor Bioaerosol Concentrations with ATP Bioluminescence Assay Using an Electrostatic Rod-Type Sampler
Source: PLoS One. 2015 May 7;10(5):e0125251. doi: 10.1371/journal.pone.0125251 (PMC4423956; doi:10.1371/journal.pone.0125251)
Supplement: S4 Information — (DOCX) [file pone.0125251.s004.docx]

**Laboratory test of bioaerosol detection**

**
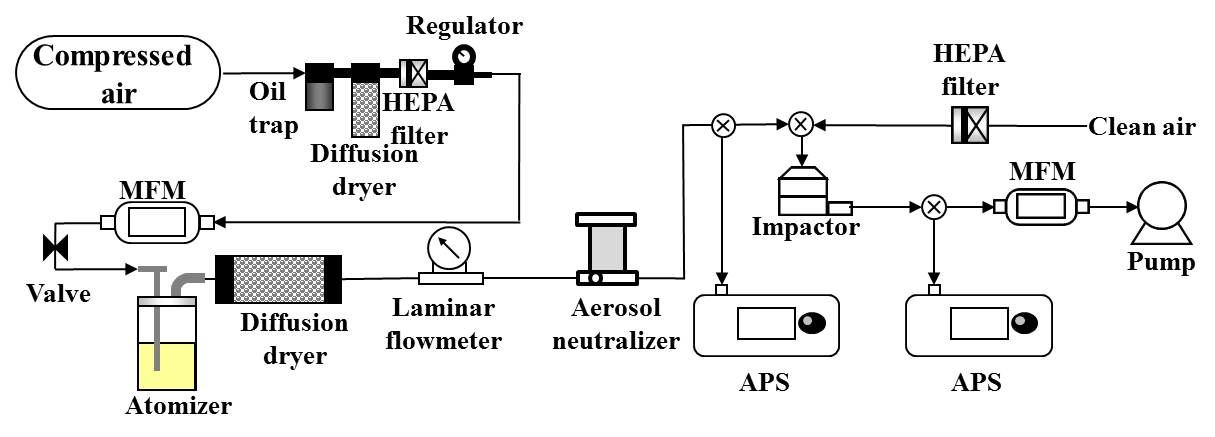
Fig. C. Schematic diagram of the experimental setup for the collection efficiency of the Andersen impactor.**

Fig. S4 shows the experimental setup for the collection efficiency of Andersen impactor. A sixth stage of Andersen impactor with a cutoff diameter of 650 nm was used with aerosolized *S. epidermidis*. The aerodynamic diameter of aerosolized *S. epidermidis* was about 800 nm. The particle concentrations were measured by APSs at the inlet and the outlet of Andersen impactor. The collection efficiency of Andersen impactor was defined below,

$\eta_{i}=1-\frac{N_{outlet}}{N_{inlet}}$ (M) where $N_{outlet}$ and $N_{inlet}$ are the number concentration of particle at the outlet and the inlet of Andersen impactor, respectively.
